# Microenvironment engineering of supported metal nanoparticles for chemoselective hydrogenation

**DOI:** 10.1039/d2sc04223a

**Published:** 2022-11-02

**Authors:** Maodi Wang, Qihua Yang

**Affiliations:** Key Laboratory of the Ministry of Education for Advanced Catalysis Materials, Zhejiang Key Laboratory for Reactive Chemistry on Solid Surfaces, Institute of Physical Chemistry, Zhejiang Normal University Jinhua 321004 China qhyang@zjnu.cn; State Key Laboratory of Catalysis, Dalian Institute of Chemical Physics, Chinese Academy of Sciences 457 Zhongshan Road Dalian 116023 China

## Abstract

Selective hydrogenation with supported metal catalysts widely used in the production of fine chemicals and pharmaceuticals often faces a trade-off between activity and selectivity, mainly due to the inability to adjust one factor of the active sites without affecting other factors. In order to solve this bottleneck problem, the modulation of the microenvironment of active sites has attracted more and more attention, inspired by the collaborative catalytic mode of enzymes. In this perspective, we aim to summarize recent advances in the regulation of the microenvironment surrounding supported metal nanoparticles (NPs) using porous materials enriched with organic functional groups. Insights on how the microenvironment induces the enrichment, oriented adsorption and activation of substrates through non-covalent interaction and thus determines the hydrogenation activity and selectivity will be particularly discussed. Finally, a brief summary will be provided, and challenges together with a perspective in microenvironment engineering will be proposed.

## Abstract 2

Insights on microenvironment engineering for metal nanoparticles using porous materials enriched with organic groups and how it determines the hydrogenation performance through non-covalent interaction are highlighted.

## Graphical abstract

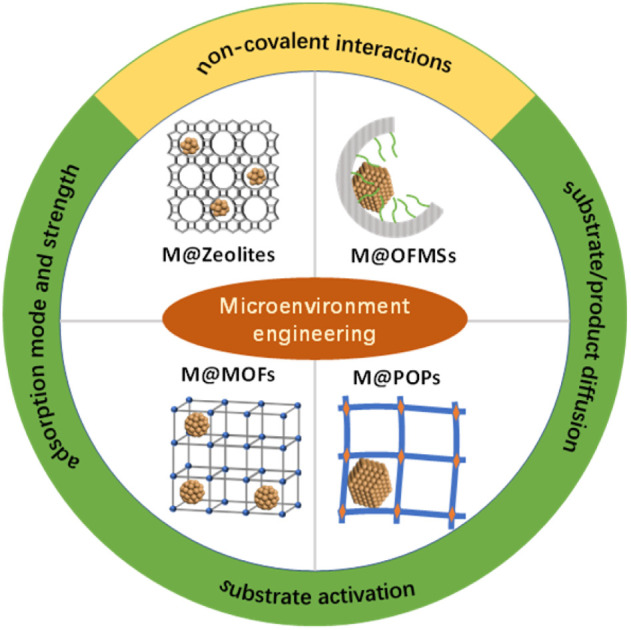

## 1. Introduction

Catalytic hydrogenation technology using supported metal nanoparticles (NPs) as catalysts has not only been widely used in petrochemical and petroleum refining, but has also been continuously developed and applied in the fields of fine chemicals and pharmaceuticals.^1–5^ Although catalytic hydrogenation technology has been developed for decades, new challenges continue to emerge due to the increasing complexity of substrates used for hydrogenation. Enormous efforts have been continually devoted to optimizing the catalytic performance of supported metal NPs.

Selectivity is becoming more and more important in view of energy saving and atomic economy. Selective hydrogenation depends on the regulation of the adsorption strength and configuration of substrates and intermediates over the active sites.^1,6^ Generally, the catalytic performance of supported metal NPs is regulated through the modulation of the electronic and geometric structures of metal sites.^7–11^ However, a trade-off between activity and selectivity was often observed in selective hydrogenation with supported metal NPs, mainly due to the inability to adjust one factor of metal NPs without affecting other factors.^1,9,12^ There is a growing recognition that the specific physicochemical microenvironment surrounding metal sites is equally important,^13–16^ as more and more studies have demonstrated that the microenvironment surrounding metal sites plays a significant role in assisting the regulation of the adsorption behaviors of substrates over metal sites through weak interaction or even provides binding sites beyond metal NPs. It should be noted that such a recognition is rooted in the understanding of the work modes of enzymes.^14,17,18^ Notably, the influence of the microenvironment on catalysis has a substantial difference with metal–support interaction, which generally modulates the catalytic performance through inducing the electronic effect,^19,20^ geometric effect^21–23^ and strong metal–support interaction^11,24–27^ of metal NPs, whereas in biological systems, enzymes, such as metalloenzymes, could catalyze chemical transformations with incredible efficiency through the elegant cooperation of metal sites and protein frameworks.^28–30^ Specifically, the binding pocket, constructed by amino acid residues around metal sites, assists in the recognition, enrichment, pre-organization and activation of substrates *via* non-covalent interactions, such as hydrogen bonds, π–π interactions, hydrophobic interactions and electrostatic interactions.^18,31^

Currently, biomimetic man-made catalysts possessing these functions have been achieved through the construction of a pocket-like microenvironment *via* assembling of functional ligands^32–37^ on the surface of metal nanoparticles (NPs) or confining of metal NPs in the nanospace of porous materials.^38,39^ These approaches are borrowed not only from enzymes but also from more developed molecular catalysts, in which organic ligands are employed to regulate the electronic structure of metal sites and also the coordination sphere.^40^ Very recently, it was reported that a C_60_-capped Cu/SiO_2_ efficiently catalyzed the hydrogenation of dimethyl oxalate at ambient pressure and relatively low temperature (180 °C to 190 °C),^41^ which is ascribed to the electron buffer effect of C_60_ between CuO and Cu^+^. Microenvironment engineering has been emerging as a valuable protocol for the rational design of catalysts, molecular understanding of catalytic mechanisms and potentially bridging heterogenous catalysis, homogenous catalysis and enzymatic catalysis.

Recent advances in the surface modification of metal NPs with organic ligands have been summarized in several reviews,^32,42–45^ and this perspective will mainly focus on metal NPs confined in porous materials. It will be started with a brief introduction to the synthesis and characterization of metal NPs with a well-defined microenvironment using porous materials as supports. Next, the catalytic performance of metal NPs modulated by weak interactions in their microenvironment will be elucidated case by case in chemoselective hydrogenation. Finally, a brief summary will be provided, and challenges together with a perspective in the microenvironment engineering of metal NPs will be proposed.

## 2. Host materials generally used to modulate the microenvironment of metal NPs

Microenvironment engineering not only assists in the improvement of the catalytic performance of supported metal NPs, but also provides a platform for the understanding of catalytic mechanisms at the molecular level. Metal NPs are generally embedded in a nanopore, considering that most of the supports used for metal NPs have porous structure. In this case, it is very important to differentiate the influence of the microenvironment from the pore confinement effect. The pore confinement effect is a generalized concept and defined by Bert *et al.* as the influence of external activity on an active site.^14^ It is mainly related to the geometric effect of a nanopore, electrostatic interactions in a nanopore, the surface properties of the pore surface, and so on. For metal NPs embedded in a porous material, these effects are always entangled together, which leads to the microenvironment effect being ignored for a long time. In this perspective, the microenvironment effect is a more specific one and we define it as the influence of non-covalent interactions between substrates and supports on the catalytic performance of supported metal NPs. Also, the examples in this perspective were carefully chosen to avoid the interference of the electronic effect and others.

The catalytic performance of metal NPs is very sensitive to the supporting matrix which may change the properties of supported metal NPs through charge transfer, metal–support interaction and so on.^46,47^ These entangled parameters make it difficult to get insight into the determining factors that govern the catalytic performance of metal NPs. Fortunately, porous materials with tailorable structures and compositions have surged over the past decades, which provides an ideal platform for the regulation of the microenvironment surrounding metal sites with near atomic-level precision. Generally, zeolites, organo-functionalized mesoporous silicas (OFMSs), metal–organic frameworks (MOFs) and porous organic polymers (POPs) are used as host materials to provide a well-defined microenvironment for metal NPs.

**Fig. 1:**
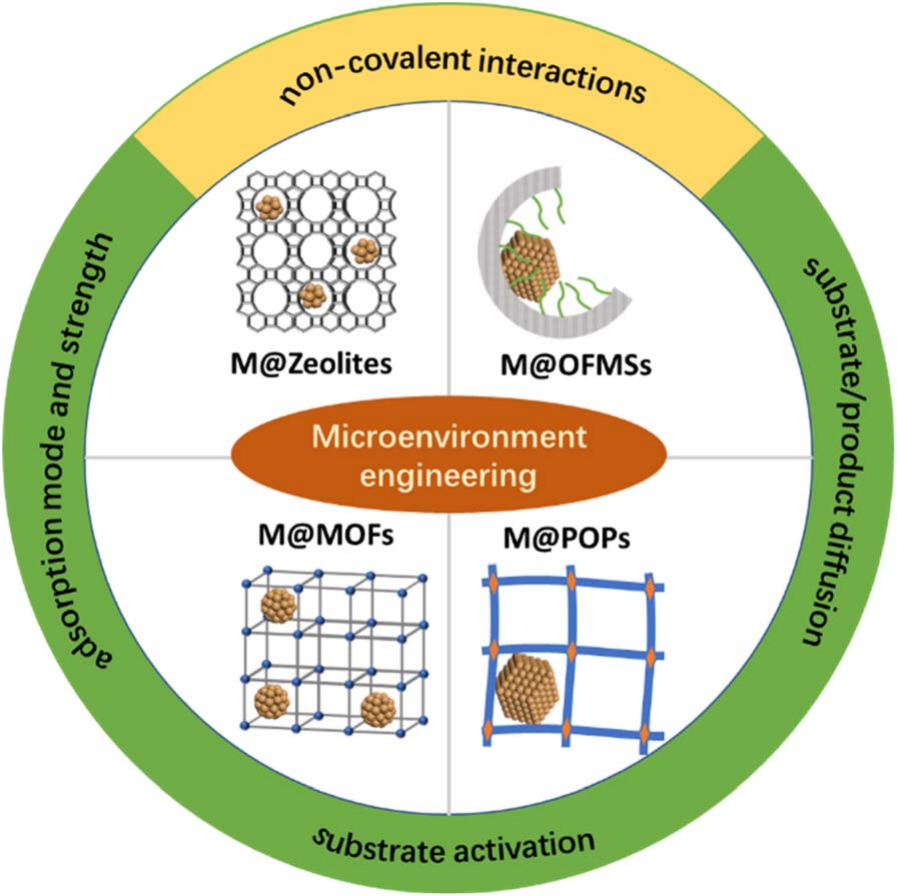
Illustration of the microenvironment modulation of metal NPs in different host materials.

Zeolites are crystalline microporous materials and have been widely used in the fields of adsorption, catalysis and ion exchange.^48,49^ Metal NPs embedded in the matrix of zeolites generally have molecular sieving properties,^50,51^ which are inherited from zeolites. Furthermore, the unique surface acid sites of zeolites can change the selectivity of metal NPs in selective hydrogenation *via* assisting in H_2_ spillover.^52^ Metal cation exchange, *in situ* encapsulation or bottle-around-ship methods have been developed to confine metal NPs within the channels of zeolites (Fig. 1).^53^

Post-grafting or co-condensation was widely employed for the preparation of OFMSs.^54,55^ By grafting or one-pot co-condensation methods, a large number of small organic molecules could be covalently anchored on the surface or in the pore wall of silicas. Besides small organic molecules, ionic liquid (IL) was also grafted on silicas for the stabilization of metal NPs.^56–60^ Leitner *et al.* demonstrated the versatility of this molecular approach for the preparation of multifunctional catalysts.^56^ Moreover, polymers could also be incorporated in the nanopore of mesoporous silicas by dispersing them on mesoporous silicas or by *in situ* polymerization in mesoporous silicas.^61^ For example, triphenylphosphine (PPh_3_) can be cross-linked in the nanocages of FDU-12 through an *in situ* solid-state polymerization method.^7,62^ Composite materials with a uniformly distributed polymer in the nanocage, a large pore size, and an ordered porous structure have different properties in comparison with bulk polymers. The nanopores of OFMSs with desired functional groups can be regarded as man-made binding pockets to mimic the functions of enzymes *via* the modulation of the weak interaction with substrates. Different strategies (wet impregnation method,^7,63,64^ one-pot sol–gel method^65,66^ and layer-by-layer assembly method^67,68^) can be used to confine mono- or multi-metal NPs in the nanopores of OFMSs.

MOFs are known as porous coordination polymers, featuring highly ordered metal nodes and organic linkers.^69,70^ The nodes of MOFs composed of metal ions or clusters commonly act as Lewis acid sites after the removal of coordinated solvent molecules.^71,72^ Thus, it's predictable the non-spectator role of MOFs in catalysis. For example, coordinatively unsaturated nodes may accept lone electron pairs from the polar moiety of substrates to promote selective activation.^73,74^ Methodologies have been well established over the past two decades to enable the preparation of isostructural MOFs composed of a wide range of metals in diverse oxidation states.^71^ When MOFs cannot be directly prepared, post-synthesis modification would be an alternative approach to further enrich the functions of MOFs while maintaining the topological structures of parent MOFs.^75–78^ Altogether, MOFs would be ideal host materials for the microenvironment regulation of metal NPs. Currently, metal NPs can be precisely loaded in different locations of MOFs using strategies such as solution impregnation, “bottle around the ship” and one-pot synthesis methods, which have been summarized in detail in recent reviews.^71,79,80^

As a class of covalently bonded materials, POPs have attracted significant attention due to their high BET surface areas and permanent porous structures.^81–83^ Compared with OFMSs and MOFs, POPs are more flexible in composition because of a broader range of applicable building blocks.^84,85^ Covalent organic frameworks (COFs) are crystalline POPs. Generally, the composition and porous structure of POPs/COF may have profound influence on the physicochemical environment of confined metal NPs. Metal NPs can be confined in POPs/COFs through a wet impregnation method or the self-assembly of POPs/COFs over the pre-synthesized metal NPs.^86–88^ In addition to these two strategies, Fischer's group developed a gas-phase infiltration method to trap organometallic precursors inside the cavity of COF-102. After the photodecomposition of precursors, monodisperse Pd NPs (2.4 ± 0.5 nm) were evenly distributed in COF-102.^89^

## 3. Regulation of the substrate/product diffusion

The enrichment of substrates from bulk solution to metal NPs and the fast diffusion of reaction products may remarkably accelerate the reaction rate and product selectivity.^90–92^ The surface wettability of the porous matrix surrounding metal NPs should be modified according to the properties of substrates. The enhanced activity of metal NPs on hydrophobic supports has been well investigated. Herein, we only give the several examples of metal NPs embedded in the porous matrix.

**Fig. 2:**
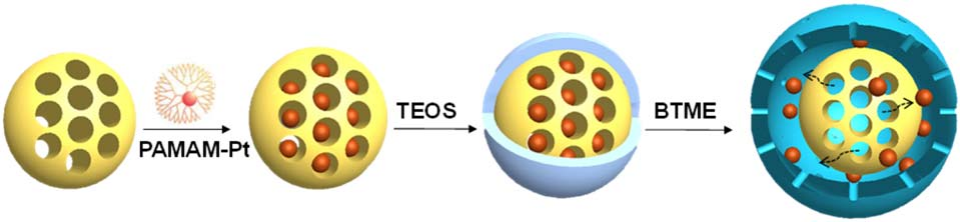
Illustration of the preparation of Pt@SiO_2_ nanoreactors through the encapsulation of PAMAM-Pt. Reproduced with permission from ref. 93. Copyright (2015) Wiley-VCH.

A silica-based yolk–shell nanoreactor confined with predefined Pt NPs (2 nm) was prepared by the adsorption of PAMAM-Pt (PAMAM-G4-OH: poly(amidoamine), 4 generation, functionalized with terminal –OH groups) onto mesoporous silica spheres with different surface hydrophobicity and hydrophilicity (Fig. 2).^93^ After that, yolk–shell nanospheres were formed by an organosilane-facilitated selective etching method. Pt@SiO_2_, Pt@SiO_2_-C_3_ and Pt@SiO_2_-Ph were prepared using mesoporous SiO_2_, propyl-functionalized mesoporous silicas (SiO_2_-C_3_) and phenyl-functionalized mesoporous silicas (SiO_2_-Ph) in the initial adsorption process, respectively. PAMAM-Pt NPs (2.1 nm) confined in yolk–shell nanostructures are much more active than the unconfined ones in the hydrogenation of cyclohexene, nitrobenzene and 4-nitrophenol, implying the positive encapsulation effect. In addition, the turnover frequency (TOF) of Pt@SiO_2_-Ph is much higher than that of Pt@SiO_2_ in the hydrogenation of lipophilic cyclohexene and nitrobenzene, but a reversed tendency was observed in the hydrogenation of hydrophilic 4-nitrophenol, which is ascribed to the fast diffusion of lipophilic and hydrophilic substrates through Pt@SiO_2_-Ph and Pt@SiO_2_, respectively.

**Fig. 3:**
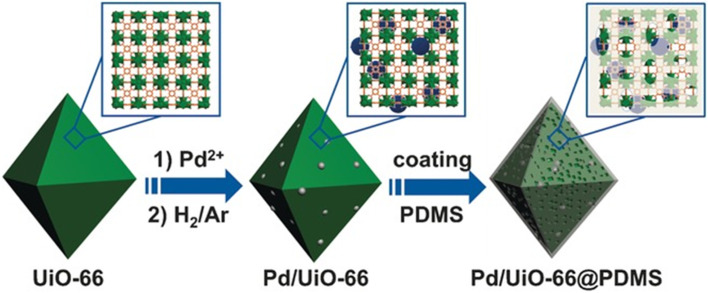
Illustration of the preparation of Pd/UiO-66@PDMS. Reproduced with permission from ref. 94. Copyright (2016) Wiley-VCH.

Jiang *et al.* reported the hydrophobic modification of Pd/UiO-66 through coating a layer of PDMS (polydimethylsiloxane) on its surface (Fig. 3).^94^ When the Pd/UiO-66@PDMS composite catalyst was tested in the hydrogenation of styrene, it took 65 min to achieve the full conversion. In comparison, a much longer time of 255 min was demanded for Pd/UiO-66. Such a promotion effect was also observed in the hydrogenation of cinnamaldehyde and nitrobenzene. Characterization results suggested that no obvious change in the size, electronic structure and accessibility of Pd NPs was observed before and after PDMS coating whereas the water contact angle significantly increased from 25° to 140° after the coating of PDMS on the Pd/UiO-66 surface. These results revealed that the surface hydrophobicity of the catalyst enhanced the affinity for hydrophobic substrates, leading to higher activity. Tang and co-workers developed another approach employing CMPs (conjugated micro- and mesoporous polymer) as the coating layer for increasing the surface hydrophobicity of MIL-101@Pt (MIL-101@Pt@FeP-CMP).^95^ Again, enhanced activity was observed in the hydrogenation of cinnamaldehyde.

**Fig. 4:**
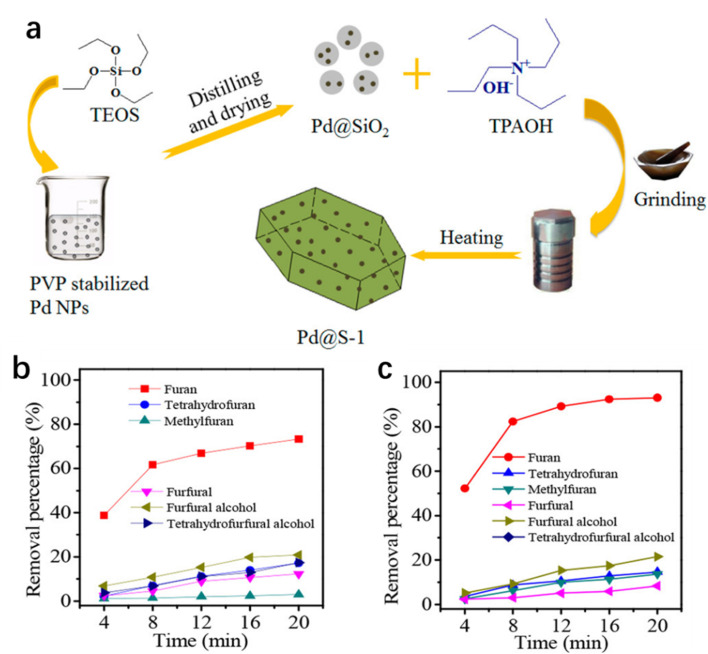
(a) Illustration of the preparation of Pd@S-1. Reproduced with permission from ref. 96. Copyright (2016) American Chemical Society. The removal percentage of various molecules over (b) Pd@S-1 and (c) Pd@S-1-OH-10 (error bars are not provided in the original figure). Reproduced with permission from ref. 97. Copyright (2018) American Chemical Society.

Besides the enrichment of substrates, the rapid desorption of the product may not only liberate the active sites for the next catalytic cycle but also favor the generation of the specific product. Xiao, Wang and co-workers reported the preparation of a core–shell structured Pd@S-1 in two steps (Fig. 4a):^96^ (1) the ship-around-bottle method was used to embed polyvinylpyrrolidone-stabilized Pd NPs in amorphous silicas to give Pd@SiO_2_; (2) Pd@SiO_2_ was crystallized into an MFI zeolite framework under solvent-free conditions. After calcination to remove the organic template, Pd@S-1 was obtained. Pd@S-1 is active in the hydrogenation of benzaldehyde (BA), but it is completely inactive with 3,5-isopropylbenzaldehyde (DPBA) as the substrate. This phenomenon is assigned to the molecular sieving effect because the molecular diameter of DPBA is larger than the micropore size of S-1, making DPBA molecules difficult to access Pd NPs (6.9 nm) through microporous channels. In the hydrogenation of furfural, Pd@S-1 gave a furan selectivity as high as 98.7%, much higher than the furan selectivity (5.6%) obtained over Pd/S-1 (Pd supported on S-1). The results of theoretical simulation and FT-IR show that the micropore of S-1 zeolite tends to adsorb the furan molecule much weaker than the other molecules, such as furfural, furfuryl alcohol, tetrahydrofuran, tetrahydrofurfuryl alcohol, and methylfuran. The extraordinary high furan selectivity of Pd@S-1 is attributed to the faster diffusion of furan molecules through the zeolite micropores than the other molecules. The catalytic activity could be further improved through the functionalization of Pd@S-1 with silanol groups (Pd@S-1-OH) in the hydrogenation of furfural to furan,^97^ which was because the hydrophilic surface of Pd@S-1-OH favored the adsorption of furfural but promoted the desorption of furan (Fig. 4b and c).

The fast mass diffusion plays an important role in accelerating the reaction rate during catalysis. However, the microporous structure of zeolites seriously constrains the mass transfer. In order to promote the mass transfer and eliminate the diffusion constraints, two strategies have been demonstrated to be effective: (1) the construction of zeolite with a mesoporous/microporous hierarchical structure.^98,99^ (2) The preparation of zeolite nanosheets to decrease the diffusion length.^100,101^ Though big advances have been made in enhancing the catalytic performance of zeolites by using the above two strategies, embedding metal NPs in hierarchical mesoporous/microporous structured zeolites or in zeolite nanosheets has not been extensively investigated. An efficient method should be developed to embed metal NPs in the matrix of hierarchical mesoporous/microporous structured zeolites or zeolite nanosheets.

## 4. Modulation of the substrate adsorption

The catalytic activity and selectivity of metal NPs largely depend on the adsorption strength and modes of substrates on the surface of metal NPs. In addition to the influence of the intrinsic properties of metal NPs, the adsorption behaviors of substrates can also be greatly affected by the structure and physical/chemical environment of supports. For metal NPs embedded in microporous materials, substrates with a molecular size larger than the pore size of the support are only allowed to adsorb on the metal surface with minimum space occupation, such as end-on adsorption mode. This specific adsorption mode always leads to greatly promoted product selectivity. For example, Xiao and co-workers reported that dichlorination is almost completely avoided in the hydrogenation of 4-nitrochlorobenzene with Pd@beta as the catalyst and severe dichlorination was observed over Pd/C, Pd/TiO_2_, Pd/Al_2_O_3_, and Pd/SiO_2_,^102^ which is related to the end-on adsorption of 4-nitrochlorobenzene on Pd@beta due to the restriction of the microporous structure of zeolite. Similarly, Xing *et al.* also reported that the selectivity to aromatic alcohols is almost 100% and 76% respectively over Y-encapsulated Pt NPs (Pt@Y, 1.9 nm) and Pt NPs supported on zeolite Y (Pt/Y, 2.35 nm) in the hydrogenation of aromatic ketones. Though the size effect cannot be completely excluded, the big difference in selectivity can only be explained by the end-on adsorption of aromatic ketones on the Pt surface.^103^

Apart from the porous structure, the physical/chemical environment of supports also has a deep influence on the adsorption behaviors of substrates through weak interactions, thus greatly boosting the catalytic performance of supported metal NPs. For example, oxygen vacancies on redox metal oxides can preferentially interact with the oxyphilic moiety of the substrate,^104,105^ the carbon/graphene can form π–π interactions with aromatic compounds,^106^ and acid/base supports can form electrostatic interactions with basic/acidic compounds.^92^ Because the above related studies could be referred in other excellent reviews,^1,91,107^ we mainly focused on porous supports in this perspective.

Chen and co-workers reported that Pd NPs encapsulated within the nanocages of amine-functionalized MOFs (Pd@NH_2_-UiO-66) displayed a high catalytic activity and exclusive selectivity to 2,3,5-trimethylhydroquinone in the hydrogenation of 2,3,5-trimethylbenzoquinone.^108^ Under similar reaction conditions, Pd NPs supported on NH_2_-UiO-66 (Pd/NH_2_-UiO-66) and encapsulated within UiO-66 (Pd/UiO-66) delivered relatively low catalytic activity. *Ab initio* molecular dynamics (AIMD) simulations suggested that the superior catalytic performance originated from the energetically favorable adsorption of 2,3,5-trimethylhydroquinone facilitated by the amine groups of MOFs, mostly arising from hydrogen bonds.

**Fig. 5:**
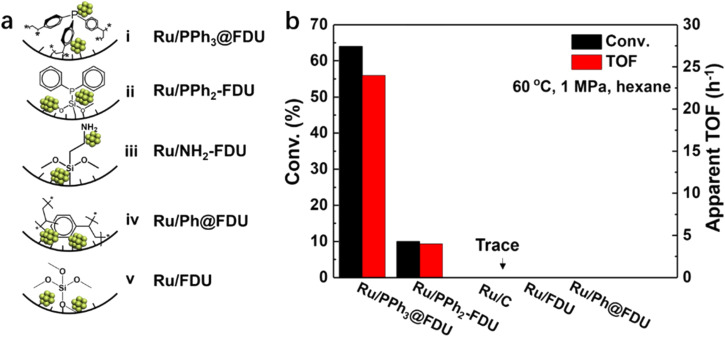
(a) Schematic diagram of Ru NPs in nanocages of FDU modified with and without organic ligands, and (b) the catalytic performance of Ru NPs in the hydrogenation of benzoic acid (error bars are not provided in original figure). Reproduced with permission from ref. 62. Copyright (2019) Wiley-VCH.

An appropriate adsorption strength between the substrate and the catalyst would be favorable in catalysis. Functional groups surrounding metal NPs may assist in the regulation of the interaction between the substrate/intermediate and the catalyst. Recently, our group prepared a series of Ru catalysts with PPh_3_, ethyldiphenylphosphine (PPh_2_), phenyl groups (Ph) and –NH_2_ in the vicinity of Ru NPs (*ca.* 2.5 nm), respectively (Fig. 5a).^62^*In situ* FT-IR of CO adsorption and XPS results indicated that the electronic density of Ru NPs followed the order of Ru/PPh_3_@FDU > Ru/NH_2_-FDU > Ru/PPh_2_-FDU > Ru/Ph@FDU & Ru/FDU, which was well matched with their catalytic activity in the hydrogenation of aromatic substrates (benzene, toluene, and trifluorotoluene), suggesting that the negatively charged Ru was beneficial for the hydrogenation of aromatic rings. However, this was not the case for the hydrogenation of benzoic acid, in which the TOFs decreased in the order of Ru/PPh_3_@FDU > Ru/PPh_2_-FDU > Ru/FDU > Ru/Ph@FDU & Ru/NH_2_-FDU (Fig. 5b). This result excludes the influence of electronic structure on the catalytic performance of Ru NPs. Density functional theory (DFT, the projector augmented-wave pseudopotential method with Perdew–Burke–Ernzerhof (PBE) exchange–correlation functional and a plane-wave cutoff energy of 400 eV was adopted.) calculations revealed that the phosphine ligands around the Ru NPs could interact with –COOH of benzoic acid with a suitable adsorption strength, leading to the oriented adsorption of benzoic acid on the Ru surface to promote the activity. The low activity of Ru/NH_2_-FDU is related to the sluggish desorption of products because of the strong interaction between –NH_2_ and benzoic acid, while the weak interaction of Ru/FDU with benzoic acid failed to induce the oriented adsorption of benzoic acid on the Ru surface.

For the selective hydrogenation of a substrate bearing multiple functional groups, the oriented adsorption of the substrate through a specific mode is essential for a desirable selectivity. In this regard, the microenvironment surrounding metal NPs may induce the oriented adsorption of substrates on metal NPs. Li *et al.* reported the preparation of Pt/MOFs@MOFs (Pt, 3.0 nm) nanocomposites through the homoepitaxial growth of the MOF layer on the surface of Pt/MOFs for the hydrogenation of cinnamaldehyde.^109^ The π–π stacking between the phenyl moiety of cinnamaldehyde and the aryl linkers of MIL-100 as well as the microporous windows of MOF layers strongly restricted the access of cinnamaldehyde to Pt NPs. As a result, cinnamaldehyde was adsorbed on Pt NPs with the terminal C=O pointing forward, leading to a greatly improved selectivity to cinnamyl alcohol from 55% to 96%.

Leitner’ group reported the rational design of Rh NPs (0.9 ± 0.1 nm) on molecularly modified SiO_2_ supports (Rh@Si–R).^110^ In the selective hydrogenation of fluoroaromatics, the selectivity to fluorocyclohexanes increased significantly along with the increase of the length of the alkyl chain grafted on SiO_2_. This is mainly because the hydrophobic microenvironment around metal NPs favors the hydrodefluorination pathway. A similar promotion effect was also observed in the selective hydrogenation of halogenated nitrobenzene by using hydrophobic Pd@MIL-101-NH_2_ prepared *via* grafting with hydrophobic perfluoroalkyls.^76^

Besides the oriented adsorption of substrates, the stabilization of particular intermediates or products may facilitate the production of targeted products. Rh@S-MIL-101 (Rh, 2.35 ± 0.9 nm) was prepared for the hydrogenation of phenol, and *a* > 92% selectivity to cyclohexanone was achieved at almost full conversion.^111^ This high selectivity was caused by the interaction between the carbonyl groups of cyclohexanone and the Lewis acidic sites of Cr(iii), thus inhibiting the over-hydrogenation to cyclohexanol.

Furthermore, substrates may be adsorbed on the sites beyond metal NPs, thus leading to a different adsorption mode. For example, Tang *et al.* reported the employment of MOFs as a selectivity regulator for the hydrogenation of unsaturated aldehydes with metal nodes as the binding sites for C=O groups.^74^ In the hydrogenation of cinnamaldehyde, bare Pt NPs afforded a selectivity of 18.3% to cinnamyl alcohol. After loading Pt NPs (2.8 nm) on MIL-101 (MIL-101@Pt), a much higher selectivity of 86.4% was achieved. FT-IR results confirmed the selective adsorption of cinnamaldehyde on MIL-101 through C=O groups because of the obvious red-shift of C=O vibration together with an unchanged C=C vibration. The calculation results indicated that the preferential adsorption of C=O over the unsaturated metal sites in MOFs rendered the hydrogenation of C=O thermodynamically favorable.

## 5. Activation of substrates

**Fig. 6:**
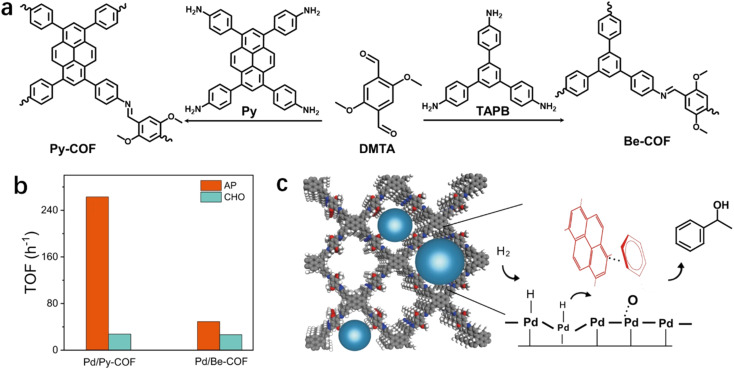
(a) Synthesis of Py-COF and Be-COF, (b) hydrogenation activity of AP/CHO over Pd/Py-COF and Pd/Be-COF (error bars are not provided in original figure), and (c) schematic diagram of Pd NPs confined in Py-COF and the proposed reaction mechanism of AP hydrogenation on Pd/Py-COF. Reproduced with permission from ref. 113. Copyright (2022) Springer Nature.

The substrates accumulated in the nanospace of porous materials may be aligned or packed orientationally through non-covalent interactions, leading to the pre-organization of substrates, which may facilitate the stabilization of substrates or intermediates. As a result, the activation barrier may be reduced. As suggested by Stoddart,^112^ a subtle difference in the energy barrier can lead to a substantial kinetic preference for a specific pathway. Recently, our group employed imine-linked COFs, Py-COF and Be-COF, with different conjugation skeletons for the immobilization of Pd NPs (Fig. 6a).^113^ Though Pd NPs (∼1.7 nm) on Py-COF and Be-COF have almost identical geometric and electronic structures, Pd NPs embedded in a pyrene-containing COF (Pd/Py-COF) were *ca.* 3 to 10-fold more active than those in Be-COF without pyrene in the hydrogenation of acetophenone (AP) and a series of polar groups of aromatic compounds. However, the promotion effect was not observed in the hydrogenation of cyclohexanone (CHO) (Fig. 6b). These results firmly demonstrated that the geometric and electronic effects were not the origin of different activities in AP hydrogenation. DFT calculations (the interaction energies were calculated at the M06-2X/def2-SVP level by including basis set superposition errors at the PBE0-D3/def2-SVP optimized structures) revealed a stronger interaction energy between Py-COF and AP, which originated from the π–π interaction between aromatics and the planar conjugated pyrene ring. The π–π interaction between Py-COF and AP was also confirmed using FT-IR and ^1^H NMR. Kinetics experiments revealed a lower apparent activation energy for the hydrogenation of AP over Pd/Py-COF. The calculated energy profiles also revealed a lower hydrogenation barrier in the rate-determining step, which was probably caused by the stabilization of the transition state by π–π interaction between pyrene rings and AP (Fig. 6c).

**Fig. 7:**
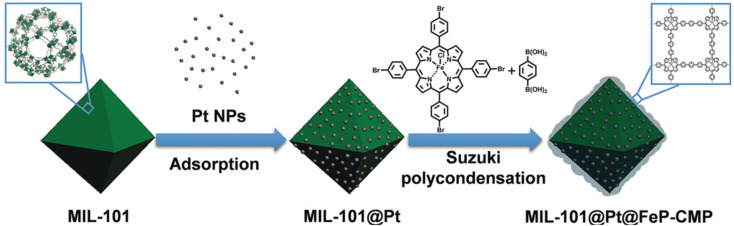
Illustration of the preparation of MIL-101@Pt@FeP-CMP. Reproduced with permission from ref. 95. Copyright (2018) Wiley-VCH.

More and more research studies discovered that the activation and hydrogenation of unsaturated bonds did not take place on metal NPs where H_2_ was dissociated, but rather on sites nearby or even far away from metal NPs.^11,114,115^ For example, coordinatively unsaturated metal sites have been recognized to be activation sites of substrates. Tang and co-workers reported the preferential adsorption and activation of C=O groups of unsaturated aldehydes over coordinatively unsaturated iron sites.^74,95^ The feasibility of this strategy was fully verified by the incorporation of iron in the vicinity of metal NPs. To be specific, metal NPs could be directly loaded in MIL-101 containing iron nodes. Iron could also be introduced to a pre-synthesized catalyst through post-modification. For example, both MIL-101(Fe)^74^ and micro- and mesoporous polymers with iron(iii) porphyrin (FeP-CMPs) (Fig. 7)^95^ were employed for the coating of a pre-synthesized MIL-101@Pt (Pt, 2.8 and 3.3 nm) catalyst to introduce iron sites. The viability of these methods was verified by the significantly enhanced selectivity and even the activity in the hydrogenation of unsaturated aldehydes. Guo *et al.* also reported that the exposed defect sites in MOFs acted as the Lewis acid sites for the adsorption and activation of both C=O groups and H_2_.^116^ The Lewis acid sites together with the appropriate mesopores greatly promoted the adsorption and activation of reactants, leading to the breaking of the trade-off relationship between selectivity and activity.

Besides the activation of polar groups, non-polar groups, such as aromatic rings, have also been reported to be activated by Lewis acid sites.^117,118^ For instance, Pd/MIL-101 with the Pd NPs (2–3 nm) surrounded by evenly distributed Lewis sites was capable of catalyzing the hydrogenation of phenol to cyclohexanone with >99.9% selectivity at room temperature and atmospheric pressure. It was shown that the coordination of phenol to Cr sites made the aromatic rings more active.^119^ Moreover, the acid–base interaction between Lewis acid and the partial hydrogenation product cyclohexanone inhibited the deep hydrogenation of cyclohexanone to cyclohexanol.

**Fig. 8:**
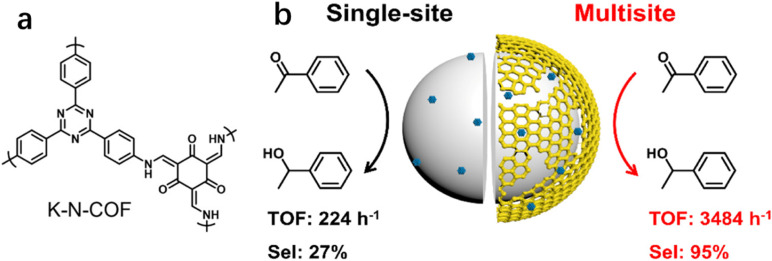
(a) Structure of K-N-COF and (b) synthesis of multisite catalyst Pt/K-N-COF/SiO_2_ (error bars are not provided in the original figure). Reproduced with permission from ref. 12. Copyright (2022) American Chemical Society.

In addition to the coordinatively unsaturated metal sites, substrates may also be activated over metal-free sites. Recently, our group reported that the selectivity to phenyl ethanol was 27.1% over Pt/SiO_2_ (Pt, 1.2 nm) in the hydrogenation of AP, but it greatly increased to 69.0% by using the physical mixture of Pt/SiO_2_ with K-N-COF (Fig. 8a) in ethanol.^12^ But the selectivity improvement was not observed when the reaction was carried out in hexane. We proposed that the promotion effect in ethanol was a result of the hydrogenation of carbonyl groups over K-N-COF using the H species dissociated on Pt/SiO_2_ through spillover. Based on these findings, a multisite catalyst Pt/Kr-N-COF/SiO_2_ (Pt, 1.6 nm) was prepared *via* integration of Pt/SiO_2_ with K-N-COF (Fig. 8b), which delivered simultaneously enhanced selectivity and activity in the hydrogenation of a series of carbonyl compounds. Notably, Pt/Kr-N-COF (Pt, 1.5 nm) presented lower activity and selectivity in comparison with Pt/Kr-N-COF/SiO_2_ due to the inefficient hydrogen spillover. This work provides an example of multisite cooperation in boosting the catalytic performance through decoupling the H_2_ dissociation sites and hydrogenation sites. The synergy effect between the organic framework and metal sites is very similar to the non-contact hydrogenation mode of reductases.^120^

Both calculation results and *in situ* diffuse reflectance Fourier transform-infrared spectra (DRIFTS) verified the adsorption of alkyne over O sites to react with OH species for the selective hydrogenation of alkyne to alkene.^121^ For example, Gong's group prepared a Pd@zeolite catalyst with Pd nanoclusters (0.8 ± 0.3 nm) confined in small-pore zeolite sodalite (SOD).^122^ In the semi-hydrogenation of acetylene, Pd@SOD exhibited an ethylene selectivity of 94.5%. The high selectivity was ascribed to the hydrogenation of acetylene over the surface OH groups of zeolites, while the adsorption of acetylene over Pd nanoclusters was impeded because of the small pore size of SOD. *In situ* DRIFTS spectra of Pd@SOD in H_2_/D_2_ confirmed the formation of OH species and its consequent reaction with acetylene.

Zhang *et al.* investigated the influence of anions on the catalytic performance of Pd NPs (1.68 ± 0.42 nm) supported on N-rich poly(ion liquid) (PIL).^123^ First, PIL was constructed based on 1,3,5-triimidazole triazine through free radical polymerization. Then, Cl^−^, PF_6_^−^ and Tf_2_N^−^ were respectively introduced in PIL through anion exchange. In the hydrogenation of nitrobenzene, the catalytic activity of as-prepared Pd NPs followed the order of Cl^−^ < PF_6_^−^ < Tf_2_N^−^. DFT calculations (B3LYP/6-311G++**) verified the strong interaction between Tf_2_N^−^ and nitrobenzene but the weakest interaction between Tf_2_N^−^ and aniline. Therefore, the authors attributed the high activity of Pd-PIL-Tf_2_N to the activation of the substrate by anions and the fast release of the product.

Actually, the activation and hydrogenation over active sites beyond metal NPs avoid the adsorption of substrates over a continuous metal surface, and thus it is not surprising to obtain high selectivity with this particular adsorption mode. Besides, metal NPs are liberated for the dissociation of H_2_ specially while avoiding the competitive adsorption of substrates. This may explain the high activity that was observed in some cases.

## 6. Conclusions and perspectives

Microenvironment engineering has become an effective strategy to improve the catalytic performance of supported metal NPs, which can improve selectivity without causing a decrease in catalytic activity. This is mainly due to the fact that it provides the possibility of independent regulation of the key parameters affecting the catalytic performance of metal NPs. Different from organic ligands, porous materials can provide a unique microenvironment for meal NPs without capping the active sites and the leaching of organic functional groups during the catalytic process is avoided. In this perspective, we have briefly introduced the main porous materials for the microenvironment engineering of metal NPs, such as zeolites, OFMSs, MOFs and POPs. The effectiveness of microenvironment engineering in improving the catalytic performance of metal NPs was verified in different selective hydrogenation reactions. The importance of the weak non-covalent interaction between the porous material and substrate is manifested in (1) enrichment of substrates through the wettability adjustment of porous materials; (2) controlling the adsorption mode of the substrate through porous materials; (3) activation of the substrate and the stabilization of the transition state through porous materials.

In this perspective, porous materials enriched with organic functional groups are chosen to illustrate the promotion effect of the non-covalent interaction between substrates, which is mainly based on the consideration of the flexibility of organic functional groups. However, it should be pointed out that the utilization of the non-covalent interaction is far beyond these materials in terms of hydrogenation reactions. One of the typical cases is carbon which has the advantages of high stability especially in H_2_O. Doping of foreign atoms (N, B, P) is an efficient strategy to modify the microenvironment of carbon.^124,125^ For example, Liu *et al.* reported that Ru (1 nm) on N-doped carbon spheres is more active than Ru on undoped carbon spheres in the hydrogenation of levulinic acid and benzoic acid, which is attributed to the enrichment of substrates around active sites through acid–base interactions of the alkaline pyridinic N on the carbon spheres and acidic substrate.^92^ Wei *et al.* reported that the activity of Pd is greatly promoted using graphene as a support in the selective hydrogenation of resorcinol attributed to the π–π interactions between graphene nanosheets and substrates.^106^ The non-covalent interaction with substrates was also found for other supports although it has been rarely applied in hydrogenation. For example, Xiang's group reported π–stacking interactions between aromatic groups and peroxide-modified titania nanosheets.^126^ These findings provide alternative possibilities in the field of hydrogenation.

Another nonnegligible factor in microenvironment engineering is the solvent effect. Generally, solvent may have an impact on the binding of reactive substrates or participate in elementary reaction steps.^127,128^ The interaction between substrates and supports may be masked when inappropriate solvents are used. For example, our group reported that the activation of carbonyl groups over K-N-COF was observed using ethanol as the solvent, while it was invalid when the reaction was carried out in hexane.^12^ This phenomenon is related to the hydrogen spillover process assisted by a protonic solvent. In another case, Liu *et al.* investigated hydrogen bonds between substrates and nitrogen-doped carbon using ^1^H NMR and a poor hydrogen bond-accepting solvent was employed to preclude the interface of solvent.^129^

It should be noted that these fundamental principles could also be applied in other reactions. For example, Xiao *et al.* reported the modification of AuPd@ZSM-5 with a hydrophobic sheath,^130^ which confined the generated peroxide in the zeolite nanoreactor. The high local concentration of H_2_O_2_ considerably enhanced the catalytic performance of AuPd in methane oxidation. Cargnello *et al.* reported the encapsulation of Pd nanocrystals within tunable microporous polymer layers,^131^ which delivered an order of magnitude higher TOF in CO oxidation. The authors interpreted this observation as the entropic stabilization of a product-like intermediate by the Lewis acid interaction of these species with the amino groups of polymer layers. More recently, Xiao, Wang, Zheng and co-workers reported that the physical mixture of CoMnC catalysts and a hydrophobic polymer greatly promoted CO hydrogenation activity through fast removal of generated H_2_O.^132^

Though selective hydrogenation has made big progress in the past decades, there are still some challenging reactions that need to be solved, such as the hydrogenation of amino acids to amino alcohols without deterioration of enantioselectivity, the hydrogenation of amides to amines without C–N bond breakage, and the direct hydrogenation of terephthalic to 1,4-dimethoxycyclohexane. Currently, the main strategy for amide/acid hydrogenation is to use Lewis acid sites to polarize the carbonyl group, however, relatively harsh conditions are still needed, which is not beneficial for retaining high chemo/enantio-selectivity.^133^ The high C=O bond energy of acids/amides is one of the challenges for amide/acid hydrogenation. Furthermore, the strong adsorption of amides/acids on the metal surface may inhibit the activation of H_2_. Microenvironment engineering enables us to mimic the collaborative catalytic mode of enzymes that have evolved over billions of years for catalytic chemical transformations. To obtain a breakthrough in acid/amide hydrogenation, microenvironment engineering will play an important role and the following concerns are proposed: (1) host–guest systems should be designed by utilizing weak interaction for more efficient activation of acids/amides. Remarkably, the dynamic structures in enzymes for specific substrates binding are rarely explored in heterogeneous catalysts. It is possible to mimic this function through the introduction of organic fragments with flexible structures in a confined space to construct a unique microenvironment for the activation of acids/amides; (2) multi-site catalysts should be designed to activate acids/amides and H_2_ on different and separated sites, which is favorable to tailor the active sites independently. The activation of acids/amides on the active sites beyond metal sites where H_2_ are activated would be a promising way to break the scaling relationship for supported meal catalysts; (3) inspirations could be drawn from not only enzymes but also other fields, such as homogenous catalysis, supramolecular and coordination chemistry. Recently, we reported a novel hydrogenation mode for efficient hydrogenation of NAD(P)^+^ to NAD(P)H through the synergy of supported metal NPs and organometallic complexes.^134^ In the synergy system, supported metal NPs are responsible for H_2_ dissociation, and the organometallic complex is responsible for further hydride transfer to NAD(P)^+^. The crossover of concepts from other fields may help to develop more efficient hydrogenation catalysts. (4) It's highly necessary to determine weak interactions quantitively especially under operation conditions, thus establishing the relationship between structure and performance beyond trial-and-error. But it remains a particular challenge in heterogenous catalysis due to the fact that weak interactions are difficult to detect and easily affected by the surrounding environment. Moreover, the dynamic behaviors on the surface may further retard the understanding of catalytic mechanisms. The advance in the high-resolution *operando* characterization techniques associated with theory calculations may assist in getting insightful information.

## Data availability

All data in this perspective were cited from other references.

## Author contributions

Q.-H. Y. conceived the topic and structure of the article. All authors reviewed and contributed to this paper.

## Conflicts of interest

There are no conflicts to declare.

## Supplementary Material
